# Supplementary material for: Impact of serum phosphate levels during CRRT on extubation failure and hospital mortality in mechanically ventilated ICU patients—A study based on the MIMIC-IV database
Source: PLoS One. 2025 Jun 18;20(6):e0323939. doi: 10.1371/journal.pone.0323939 (PMC12176230; doi:10.1371/journal.pone.0323939)
Supplement: Attachment — Submitted filename: Response to Reviewers.docx [file pone.0323939.s002.docx]

Dear Editor,

Thank you for handling our manuscript (PONE-D-24-59339) and providing constructive feedback. We have carefully addressed all reviewers’ comments as follows:

**Reviewer 1,**

**Comment 1:** In lines 46-47 it reads " However, it is imperative to monitor the loss of amino acids" - I would consider eliminating de amino acids part, since it's not within the scope of the article and, to the best of my knowledge, there is no method to monitor it on a daily basis in the critical care setting.

**Response:** We sincerely appreciate this insightful suggestion. Upon careful consideration, we agree that the mention of amino acids monitoring deviates from the primary focus of our study on serum phosphate. Additionally, as the reviewer rightly pointed out, current clinical practices in critical care settings lack standardized methods for routine amino acids monitoring. We have therefore removed the phrase "amino acids" from Line 46 (now Line 46 in the revised manuscript) and adjusted the sentence to:

_"However, it is imperative to monitor the loss of electrolytes, including phosphate, during continuous renal replacement therapy (CRRT) to avert potential imbalances."_

This modification enhances the coherence of our argument while maintaining clinical relevance. All changes have been highlighted using ‘Track Changes’ in the revised manuscript.

**Comment 2:** In lines 106-107 it reads "In this study, failure to extubate is defined as a period of less than 48 hours between the last extubation in the ICU and discharge from the ICU." - I believe the authors would like to furthur clarify their definition of "extubation failure" - it is need for re-intubation within a period of less than 48h after a planned extubation?

**Response:** We thank the reviewer for highlighting this ambiguity. To align with standard clinical definitions [1], we have revised the definition to explicitly incorporate reintubation and spontaneous breathing sustainability. The updated statement in Lines 106-107 (now Lines 105-107 in the revised manuscript) reads:

_"In this study, extubation failure is defined as the need for unplanned reintubation within 48 hours following a planned extubation attempt, or failure to maintain unassisted spontaneous breathing for ≥48 hours."_

This clarification ensures consistency with widely accepted criteria [1] and eliminates potential misinterpretation of the outcome measure. All modifications have been tracked in the revised manuscript.

Reference:

[1] Burns KEA, Wong J, Rizvi L, et al. Frequency of Screening and Spontaneous Breathing Trial Techniques: A Randomized Clinical Trial. JAMA. 2024;332(18):1808-1821. doi:10.1001/jama.2024.20631

**Reviewer 2,**

**Comment 1:** Lines 106-107: Please clarify the definition of "extubation failure": "In this study, failure to extubate is defined as a period of less than 48 hours between the last extubation in the ICU and discharge from the ICU." What is the reference for this definition?

**Response:** Please refer to the response to the second comment from the first reviewer for details.

**Comment 2:** Table 1: Please clarify what is meant by "Postoperative complication."

**Response:** We appreciate the reviewer's request for clarification. In this study, "postoperative complications" were defined as clinically significant adverse events occurring after invasive procedures or surgical interventions.Documented in the MIMIC-IV database with ICD-10 codes indicative of procedure-related complications (e.g., T82.7: Infection and inflammatory reaction due to cardiac device, implant, and graft; K91.841: Postprocedural hemorrhage of a digestive system organ or structure following a digestive system procedure). However, due to the different methods of invasive surgery or surgical intervention, they cannot be classified.

**Comment 3:** Lines 316-317: Nutritional phosphate intake could significantly influence the amount of phosphate absorbed by ICU patients. This could be a relevant factor and should be considered more thoroughly. Although it is mentioned, this aspect should be further emphasized.

**Response:** We sincerely appreciate the reviewer's valuable suggestion regarding the role of nutritional phosphate intake. While our study lacks granular data on phosphate intake quantification, we have emphasized the critical role of phosphate supplementation within ICU nutritional management protocols in the Discussion section. See lines 320-323 for details.

Attached are the revised manuscript (tracked changes and clean versions). We confirm compliance with PLOS ONE’s data policy.

Sincerely,

Liang Gao
